# Emerging magnetic resonance imaging techniques in open spina bifida *in utero*

**DOI:** 10.1186/s41747-021-00219-z

**Published:** 2021-06-17

**Authors:** Andras Jakab, Kelly Payette, Luca Mazzone, Sonja Schauer, Cécile Olivia Muller, Raimund Kottke, Nicole Ochsenbein-Kölble, Ruth Tuura, Ueli Moehrlen, Martin Meuli

**Affiliations:** 1 Center for MR-Research, University Children’s Hospital Zürich, Zürich, Switzerland; 2 Neuroscience Center Zürich, University of Zürich, Zürich, Switzerland; 3 Department of Pediatric Surgery, University Children’s Hospital Zurich, Zürich, Switzerland; 4 The Zurich Center for Fetal Diagnosis and Therapy, Zürich, Switzerland; 5 Department of Surgery, Cantonal Hospital of Aarau, Aarau, Switzerland; 6 Department of Diagnostic Imaging, University Children’s Hospital Zurich, Zurich, Switzerland; 7 Department of Obstetrics, University Hospital Zurich, Zürich, Switzerland; 8 University of Zurich, Zürich, Switzerland

**Keywords:** Magnetic resonance imaging, Diffusion tensor imaging, Foetus, Infant (newborn), Spinal dysraphism

## Abstract

Magnetic resonance imaging (MRI) has become an essential diagnostic modality for congenital disorders of the central nervous system. Recent advancements have transformed foetal MRI into a clinically feasible tool, and in an effort to find predictors of clinical outcomes in spinal dysraphism, foetal MRI began to unveil its potential. The purpose of our review is to introduce MRI techniques to experts with diverse backgrounds, who are involved in the management of spina bifida. We introduce advanced foetal MRI postprocessing potentially improving the diagnostic work-up. Importantly, we discuss how postprocessing can lead to a more efficient utilisation of foetal or neonatal MRI data to depict relevant anatomical characteristics. We provide a critical perspective on how structural, diffusion and metabolic MRI are utilised in an endeavour to shed light on the correlates of impaired development. We found that the literature is consistent about the value of MRI in providing morphological cues about hydrocephalus development, hindbrain herniation or outcomes related to shunting and motor functioning. MRI techniques, such as foetal diffusion MRI or diffusion tractography, are still far from clinical use; however, postnatal studies using these methods revealed findings that may reflect early neural correlates of upstream neuronal damage in spinal dysraphism.

## Key points

- Magnetic resonance imaging provides morphological cues about hydrocephalus development, hindbrain herniation or outcomes related to shunting and motor functioning.
- Advanced image processing, such as super-resolution reconstruction, enables a better depiction of anatomy in spina bifida.
- Diffusion tensor imaging may provide early markers of upstream neuronal damage; however, the method is still in its infancy prenatally.

## Background

Open spinal dysraphism, also termed spina bifida aperta, or myelomeningocele (cystic variant) or myeloschisis (noncystic variant), is one of the most complex and devastating nonlethal congenital malformations. Structurally, it is characterised by a posteriorly open spine, an open dura mater fused with the dermis of the surrounding skin, an open pia mater fused with the epidermis of the adjacent skin, and a non-neurulated spinal cord residing on top of the pia mater, directly facing the amniotic cavity and fluid [1].

Pathogenetically, the above anatomical configuration sets the stage for deleterious effects. According to the so called “two hit pathogenesis”, the process leading to the described abnormal architecture within the lesion is the first hit and, importantly, does not automatically cause functional problems. These arise mainly due to progressing damage to the unprotected and extremely fragile, openly exposed spinal cord tissue, particularly during the third trimester of pregnancy. Here, a combination of traumatic, mechanical, chemical, toxic, and degenerative factors finally destroys the cord tissue and cause massive functional loss [2–6]. Clinically, open spina bifida is typically associated with a core cluster of cerebral, visceral, and peripheral neural pathologies. Most patients suffer from hydrocephalus that requires an operative treatment (ventriculoperitoneal shunt or third ventriculostomy) [7, 8]. Almost all patients exhibit both severe neuropathic bladder and rectum with vexing incontinence for urine and stool, as well as, later in life, sexual dysfunction. Finally, most individuals demonstrating the classical lumbosacral lesion experience paraparesis or paraplegia with serious problems regarding independent ambulation, and many are confined to the wheelchair for life.

Therapeutically, there is a large and compelling body of experimental and clinical evidence for the efficiency of foetal surgery for open spina bifida [9]. After the Management Of Myelomeningocele Study (MOMS Trial, 2011) produced clear cut data that prenatal surgery produces significantly better outcomes than postnatal care [10]. Since this study, a report found that short-term outcomes after pre- and postnatal correction might be equivalent [11]. Nevertheless, *in utero* operative treatment became a novel standard of care for select foetuses with spina bifida [5, 12–15]. There is a certain evidence suggesting that open foetal surgery and foetoscopic spina bifida repair might result in similar short-term benefits for the newborn, while maternal obstetric outcomes appear to be better after the foetoscopic intervention [16].

While a preoperative maternal-foetal magnetic resonance imaging (MRI) investigation is considered an obligatory clinical standard before eventual foetal surgery, this technology harbours other attractive options to cast light on both structural and functional features of one of the most complex cerebrospinal malformations. Prenatal imaging has become a part of surgical planning for the depiction of the spine defect as well as associated brain abnormalities, such as Chiari-II or callosal malformations [17–19]. Findings in one of the earliest reports of its kind concluded that prenatal MRI is effective to confirm the lumbosacral skin defect in open spinal dysraphism [20]. Three decades later, technical advancements enabled a widespread use of MRI and made foetal MRI the second-line imaging modality for neural tube defects after ultrasonography [21–24]. An important step was the possibility to shorten imaging time by new sequences [25–27]. There is growing evidence for the efficiency and clinical benefit of foetal surgery in spina bifida [5, 6, 13, 14], subsequently leading to an increasing demand for prenatal imaging for pre-surgical assessment and postsurgical longitudinal follow-up.

The main purpose of our review was to make emerging and experimental MRI acquisition and processing techniques, which might not yet have been implemented in the widespread clinical practice, known to experts from diverse clinical subspecialties. We aimed to provide a critical perspective on how diagnostic and follow-up imaging in spinal dysraphism can be improved by emerging MRI acquisition, image reconstruction and quantification methods. Firstly, we reviewed studies that used morphological MRI biomarkers to characterise structural and functional neurodevelopment in spinal dysraphism and attempted to make predictions of how such insights could be utilised in prenatal diagnostic imaging. Secondly, we focused on advanced techniques to improve the anatomical resolution and utility of foetal MRI for image quantification. Third, we performed a search for articles that adapted MRI sequences to the *in utero* setting in order to quantify foetal physiology. One of the most promising and widely used MRI modality for this purpose is diffusion-weighted foetal MRI, which probes the structure of extracellular fluid spaces and depicts major neuronal bundles. In all parts of this review, case reports have been excluded from the literature search results.

## Established structural MRI biomarkers in open spinal dysraphism

Prenatal ultrasonography (US) alone yields high diagnostic accuracy in all forms of spinal dysraphism [28], yet MRI has the advantage of delivering additional anatomical as well as physiological information, such as tissue microstructure or organ function, particularly for the brain.

Foetal MRI requires no maternal or foetal sedation and can be performed within the time limits of a typical clinical imaging session. The total scan time varies between 45 and 60 min [25, 29, 30]. In combination with anatomical measurements from US, standard care MRI (fast, single shot sequences) can deliver useful predictors of outcome in spina bifida.

In an ongoing effort to find hydrocephalus predictors after prenatal repair of spina bifida, quantitative measurements were identified which are simple to perform on US or MRI. In one of the earliest works, Bruner et al. found that in foetuses who underwent prenatal surgery, ventricular size at the time of surgery and high defects were predictors for the need for ventriculoperitoneal shunting (VPs) for hydrocephalus during the first year of life [31]. An important study analysing randomised groups from the MOMS trial found a clear relationship between ventricle size and VPs requirement in both the pre- and postnatally operated cases [7]. This study did not find a correlation between the lesion level and eventual need for shunting. There is consensus on the predictive power of ventricular width, size and morphology, measured by either US or MRI [8, 32, 33].

In 16 subjects undergoing prenatal repair, Zarutskie et al. [24] found that the most important predictor of the need for postnatal hydrocephalus treatment was persistent hindbrain herniation on postoperatively acquired MRI. Further significant MRI predictors included ventricular volume, ventricular volume growth and hindbrain herniation, all exceeding the predictive power of US-based measurements. In a more recent study, the reversibility of the hindbrain herniation was found to be an important predictor of a reduced chance of VPs requirement [34]. Morphological cues measured on MRI, such as hindbrain configuration and lateral ventricle size, were found to be different between myeloschisis and myelomeningocele [35]. MRI also depicts essential features of the lesion configuration, such as the presence and size of a sac in open spinal dysraphism, which is associated with a more common occurrence of talipes [36].

As MRI is readily applicable in the clinical practice to assess the presence and degree of the hindbrain herniation [37, 38], this modality has an emerging importance in finding novel markers of VPs requirement or in assessing neurological outcomes in spinal dysraphism. Nevertheless, 30-month neurodevelopmental outcomes including motor functioning were not significantly correlated with prenatal ventricle size or VPs placement, and the absence of a sac over the lesion was the only MRI-derived parameter that was associated with walking independently [39]. Interestingly, a study found that the degree of cerebellar herniation on foetal MRI was significantly associated in seizure activity and high-risk bladder dysfunction in children with spina bifida [40]. Finding reliable imaging-based correlates of infant mental and motor outcomes remains a timely endeavour.

## Techniques improving the anatomical resolution and utility of foetal MRI

**Fig. 1:**
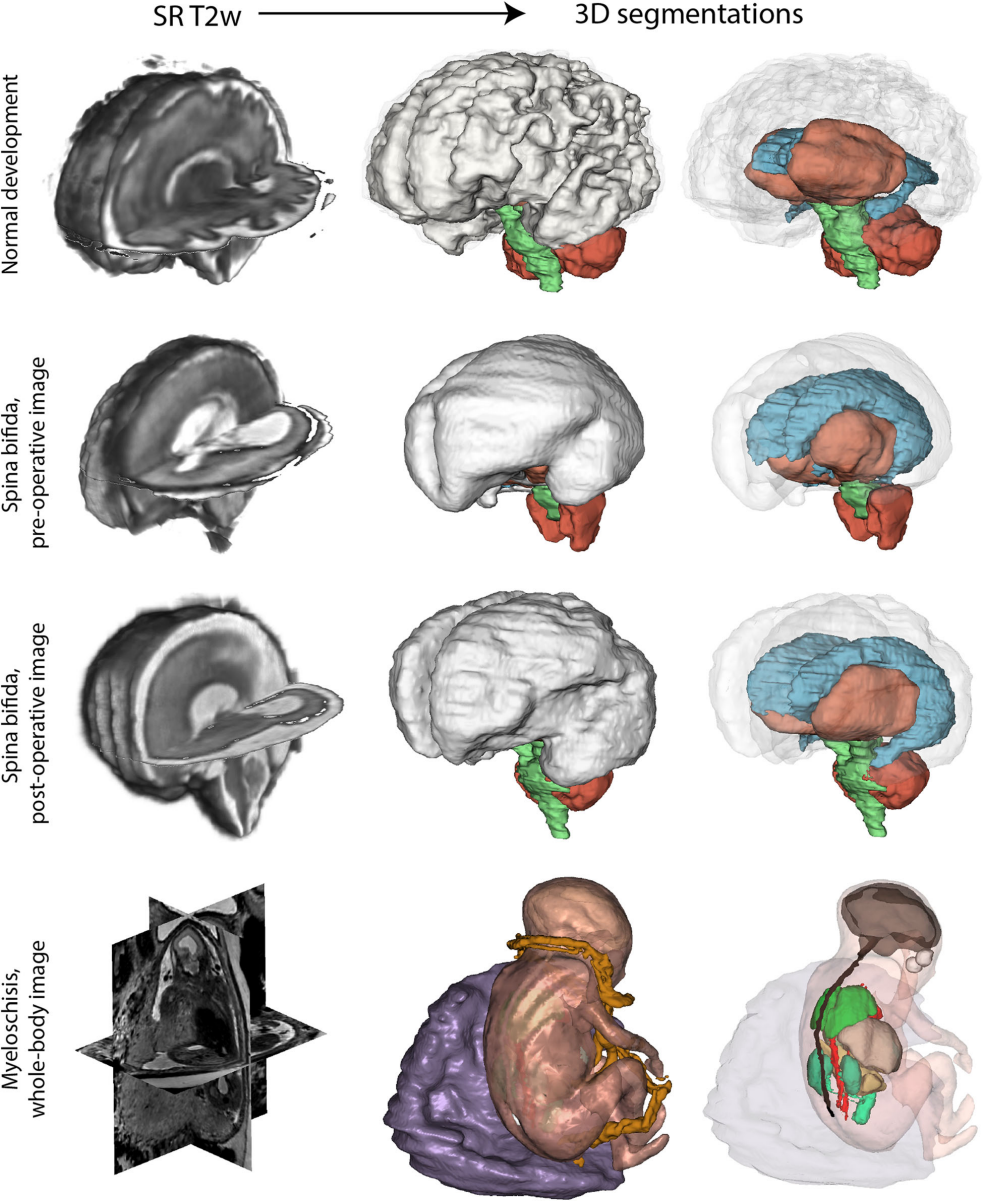
Super-resolution (SR) reconstruction of foetal MRI. For each case (normal development, pre-operative, and post-operative images of spina bifida foetus undergoing foetal surgery and myeloschisis), we demonstrate the super-resolution T2-weighted MRI (left images) and two surface reconstructions that were based on the segmentation of the reconstructed image (right images)

In the clinical practice, MRI is still limited to two-dimensional (2D) measurements. Movement artefacts often hinder the evaluation of foetal MRI, and it offers relatively poor contrast for skeletal structures. In this section, we reviewed methods that aimed to overcome these limitations. As pointed out by a study describing the inter-observer variability of essential MRI features of Chiari-II malformations, some measurements are unreliable and depend on the selection of a specific imaging plane, which is often challenging due to foetal movements [41]. More specifically, brainstem measurements were reported to be particularly unreliable in foetuses with open spina dysraphism [22]. 3D acquisition and consequent multi-plane reconstruction would theoretically solve this problem, if it was not for the foetal motion present, inducing imaging artefacts. The foetus is not sedated during the MRI; therefore, motion cannot be controlled. In order to combat motion artefacts, acquisition is done using fast MRI sequences such as the single shot fast spin-echo T2-weighted sequence, which acquires several 2D slices over a three-dimensional (3D) space. However, since foetal motion is present between the slice acquisitions, there is a distortion in the anatomy of the foetus within the image stack. In order to correct inter-slice motion and to obtain higher resolution images, super-resolution (SR) techniques are used. Many groups have been working on various SR techniques. In Slice to Volume Registration (SVR), multiple orthogonal low resolution (LR) image stacks are acquired, and motion is in part tackled by combining these multiple 2D stacks with overlapping information into a single high resolution (HR) 3D volume of the region of interest in the foetus. Various implementations of the SVR technique have been created using slice intersection motion correction [42], which directly co-aligns multiple slice stacks by matching the structures along all intersecting slice pairs in all orthogonal slices acquired [43], incorporating knowledge of the slice acquisition model based on a robust estimation [44]. Intensity matching and outlier removal have also been used [45–47]. The most common application of foetal imaging using SVR is the brain, as this method can most effectively be used for correcting motion of rigid structures, such as the brain. SR techniques allow fine structures within the brain to be imaged, and result in an overall increase in the signal to noise ratio [42]. In addition, while orthogonal planes need to be acquired, the imaging orientations do not need to be perfectly ‘in-plane’, thereby reducing the planning time during the acquisition. Preliminary use of these SR techniques has shown promise in quantifying various aspects of the foetal brain such as cortical folding patterns, volumetric and morphometric analyses for pathologies such as ventriculomegaly, and cortical plate and subplate volume growth analysis during foetal development [48–50]. We demonstrate the SR techniques in a normally developing foetus and a foetus with spinal dysraphism undergoing foetal surgery in Fig. 1.

For reconstruction of the foetal spine, internal organs and placenta, the SVR methods mentioned above cannot resolve the issue of difficult registrations due to non-rigid motion, resulting in degradation of image quality within the reconstructed volume. Therefore, a method was developed that uses non-rigid image registration and SVR (deformable SVR) which shows great promise for reconstruction high resolution images of the spine and surrounding organs in spina bifida [51]. Patch-to-volume reconstruction is also able to reconstruct a large field of view of non-rigidly deforming structures, using a parallelised patchwork optimisation (patch to volume reconstruction is used instead of SVR), SR, and automatic outlier rejection. Robust statistics are used to identify mis-registered or heavily corrupted data [52]. These HR volumes can facilitate early diagnosis of various congenital diseases and also allow for quantitative investigation of foetal development *in vivo* in spinal dysraphism [53].

Improvements in both hardware and software methods are required to overcome the movement artefacts present while imaging an unpredictably moving foetus or newborn. Since foetal structural MRI data in the setting is typically acquired using 2D sequences with repetition time in a range of seconds, spurious foetal movement affects only those slices that were acquired at the time the foetus moves, and some of the data remains usable for image analysis. As presented previously in our review, many slice-to-volume methods are feasible for tackling subject motion while at the same time improving the off-plane spatial resolution. In spina bifida imaging, foetal MRI is greatly limited by the lower spatial resolution for the assessment of the level of the lesion and to depict the myelon. Recent works demonstrated that isotropic reconstructions of the entire foetal body including the spine are possible [51], which could be used to tackle this problem. Faster image acquisition would also improve the clinical utility of foetal MRI, for example, by using compressed sensing technique [54].

Higher field strength, such as 3.0 T, potentially improves spatial resolution and increases the signal-to-noise ratio with proper sequence adaptation. Therefore, it has clinical potential for prenatal imaging in spina bifida [55]. The detection of skeletal structures is also limited in T2-weighted foetal MRI. A possible way to overcome this is to use T2*-based and modified volumetric interpolated breath-hold examination (VIBE) sequences, which were reported to be a potential alternative to US or CT imaging in detecting foetal dysplasia [56, 57]. More recently, foetal “black bone” MRI by means of susceptibility-weighted imaging (SWI) has been demonstrated to be a viable technique for evaluation of foetal spinal pathologies. Unlike conventional MRI sequences, SWI depicts the developing skeletal tissues with sufficient contrast [58] and, as suggested by the Robinson et al., would be useful for the prenatal assessment of skeletal defects. Figure 1 illustrates how whole-body HR volumes could be used for reconstructing the foetal myelon as well as other organs.

## Diffusion MRI

In the last three decades, diffusion magnetic resonance imaging (dMRI), an umbrella term for diffusion-weighted imaging (DWI) and diffusion tensor imaging (DTI), has been successfully used to characterise tissue microstructure and white matter anatomy [59]. This method is based on the sensitisation of the image to microscopic-scale diffusion-driven molecular motion. dMRI is increasingly used prenatally [60–64]. Its clinical utility in spinal dysraphism remains less established since there are only a few studies using it prenatally.

A potential application of dMRI prenatally is to characterise white matter development and upstream neuronal damage in spinal dysraphism. The neurodevelopmental sequelae in spinal dysraphism may in part stem from *in utero* injury to the developing white matter, which would lead to measurable changes in the diffusion properties of the cerebral tissue. Shrot and Soares [65] used foetal cerebral dMRI to quantify the apparent diffusion coefficient (ADC) in a cohort of open spina bifida cases. They found that the frontal and temporal lobes had lower ADC values in spina bifida cases than controls, indicating previously unknown microstructural changes in the supratentorial brain parenchyma. High spinal dysraphism (higher than L4 level) was associated with increased severity of ventriculomegaly, but no specific differences in diffusion properties were associated with the level of the spinal lesions [65]. The cerebellar ADC of foetuses with Chiari-II malformation was higher [66], indicating disrupted membrane integrity and altered neuronal tissue composition.

Fractional anisotropy, which is a DTI-derived measure of axonal density and myelin integrity, was found to be elevated in the midbrain of foetuses with Chiari II malformations and spina bifida, but not in foetuses with hydrocephalus and mild ventriculomegaly without Chiari II and no spina bifida [67]. It has been suggested that the accompanying Chiari-II malformation (present in almost all open spina bifida cases) leads to hydrodynamic changes, such as blockage of cerebrospinal fluid (CSF) drainage, which causes microstructural and tissue diffusivity changes. These hydrodynamic forces likely impact the water compartment in the cerebral white matter, leading to abnormalities in dMRI, which could potentially be used to monitor or predict hydrocephalus development [68, 69].

While foetal DTI revealed white matter diffusion abnormalities linked to spina bifida, it remains unclear whether these changes are persistent in later life and whether they are affected by surgical intervention or the anaesthesia. Studies in children and adults with spina bifida can help elucidate the consequences of the spinal abnormality on long-term neurodevelopment. Postnatal MRI revealed that patients undergoing foetal repair show white matter abnormalities when compared to controls [70]. Sanz Cortes et al. [71] investigated how tissue microstructure, measured using diffusion MRI, is affected by the prenatal operation technique. They found no significant differences in ADC values in any of the brain areas measured between the open-repair and foetoscopic-repair groups.

Postnatal findings in spina bifida point to the presence of additional white matter abnormalities that are not necessarily the consequence of altered hydrodynamic forces, but rather a result of upstream neuronal damage [72]. The supratentorial white matter is likely affected by the additive effect of CSF blockage and an upstream neuronal injury, leading to widespread microstructural abnormalities [73, 74]. dMRI studies indicate that deep grey matter structures and almost all major white matter pathways appear to be affected in spina bifida [75–77]. The abnormal findings in cerebral white matter and deep grey matter represent a possible microstructural basis for neuropsychological abnormalities in numerous studies, and open a window on using dMRI to predict later life cognitive development in spina bifida [76–79].

However, no robust data are available about the correlation of the prenatal diffusion anomalies and postnatal white matter alteration, and possible links to neurodevelopmental outcomes are yet to be investigated.

**Fig. 2:**
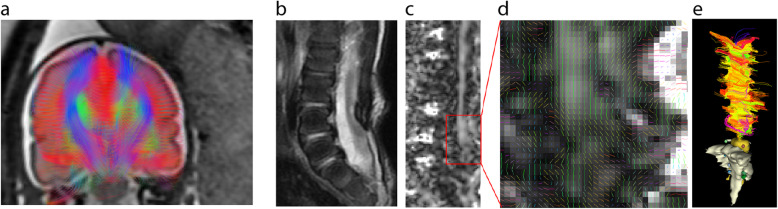
Diffusion tensor imaging in spinal dysraphism. **a** Diffusion tractography of a foetus with spinal dysraphism, 26th week of gestation. The fibre tracts (colour representing spatial orientation) have been overlaid on a coronal plane, T2-weighted MR image. **b** Sagittal T2-weighted MRI of a newborn with spina bifida, corrected prenatally, showing signs of tethered cord. **c** Sagittal fractional anisotropy image of the same myelon cross-section, demonstrating increased anisotropy in the region of the tethering. **d** Diffusion tensor principal vector orientations in the same region. **e** Diffusion tractography of the myelon

Tractography is an image processing technique that extends the application of dMRI by enabling the reconstruction of major white matter pathways [59] (Fig. 2a). It remains to be established if aberrant neuronal pathways can be detected in spinal dysraphism prenatally using tractography. A potential application of dMRI and tractography in spinal dysraphism is to characterise the microstructural properties of the spinal abnormality, including the spinal cord and its distal peripheral nerves, namely the pelvic nervous network (Fig. 2 (b–e)). The use of dMRI for the paediatric spine and pelvic nervous network is challenging and only a few studies have attempted to use dMRI in spinal dysraphism. Some of the reported paediatric cases are spina bifida patients with sacral nerve roots agenesis [80]. The authors report similar fractional anisotropy (FA) values in spina bifida patients as in controls, while mean, axial and radial diffusivity values at S1–S3 were significantly lower in patients with spinal dysraphism. Tractographic reconstruction of the sacral plexus was successful and revealed that the plexus in patients with spinal dysraphism is asymmetrical and disorganised compared to healthy controls.

There is a scarcity of studies that have explored the feasibility of tractography of the pelvis nervous network. The sacral plexus from L4 to S4 has been explored in a group of children with spinal dysraphism and in a group of healthy adult volunteer by [81]. They reported no differences between left and right sides across the subjects in terms of FA and mean diffusivity (MD) values and failed to reliably track S4 roots. To our knowledge, there is no prenatal tractography study on foetuses presenting spinal dysraphism. Prenatal dMRI studies on the peripheral nervous system raise even more challenges related to imaging artefacts because of the small calibre of peripheral nerves showing low levels of FA [82].

Unfortunately, echo-planar sequences, such as those used during dMRI/DTI, are increasingly sensitive for patient motion. They are also more prone to susceptibility artefacts, which are common near the air pockets in the bowels or near pelvic skeletal structures. This makes the quantification of diffusion anisotropy values or tractography very challenging. Recent efforts aimed to tackle this problem and used advanced reconstruction methods for scattered DTI data [83–85] or higher order spherical harmonics-based modelling [86, 87], which led to higher anatomical precision for the visualisation of white matter pathways in the foetal brain.

## ^1^H-MRS

^1^H-MR spectroscopy (MRS) is a commonly used tool to study the metabolite concentrations of tissues *in vivo*. For spinal dysraphism, this method has an emerging potential as metabolic profiling of the amniotic fluid may reflect foetal maturation. During the pathogenesis of spinal dysraphism, ischemia, anaerobic metabolism, and disruption of the neuronal membrane lead to changes in the chemical composition of the CSF and the amniotic fluid. Experiments in animals already showed promise in this direction [88].

Degradation products or other compounds in the CSF and amniotic fluid in open forms of spina bifida could open up novel avenues in a search for potential markers of neurologic dysfunctions using MRS. MRS revealed higher succinic acid and glutamine concentrations in amniotic fluid in foetuses with spinal dysraphism compared with controls [89]. Both compounds are present in cerebrospinal fluid and the higher concentration of succinic acid may hint towards a disturbance in the citric acid cycle or in the oxidative phosphorylation. Compared to controls, higher levels of lactate, choline, glycerophosphocholine, acetate, and alanine were found in the CSF of adult patients with spinal dysraphism [90], which was partially confirmed by another study focusing on tethered cord syndrome [91].

In MRS, higher magnetic field strength results in better spectral separation and higher signal-to-noise ratio. Therefore, using 3.0-T MRI for MRS would potentially increase the number of detectable metabolites at high spatial specificity.

## Conclusions

Diagnostic imaging by means of prenatal MRI is currently undergoing a notable paradigm shift from being a qualitative, second-line option to becoming a quantitative method in the realm of precision medicine. In the context of spinal dysraphism, the experimental and clinical works describing emerging techniques presented in our article represent the first steps towards this direction.

There is growing evidence that prenatal structural MRI can predict the need for VPs as well as when foetal surgery will reverse hindbrain herniation. It can also provide morphological cues for later adverse motor development, such as the presence of a sac over the spinal lesion. The greatest expectation for foetal MRI in the clinical setting is to have high spatial resolution without prolonged imaging time. However, prenatal structural MRI suffers from limitations arising as a result of unpredictable foetal movement. Despite having better soft tissue contrast, the spatial resolution of foetal MRI is lower than that of US. Resolution is improved with foetal MRI performed at 3.0-T field strength. Techniques that accelerate MRI, such as compressed sensing, would eventually be used to achieve higher resolution and higher signal-to-noise ratio. Movement artefacts could be mitigated by the post-processing software algorithms presented in our article. Super-resolution algorithms provide higher resolution reconstructions of the 3D cerebral anatomy, which, besides the possible visualisation of critical anatomical structures in open spina bifida, can be used to quantitatively search for new outcome predictors in spinal dysraphism. Further software developments, such as deep learning reconstruction as well as novel MR sequences with more specific tissue contrast, will allow for the 3D reconstruction of the spinal lesion and the surrounding soft tissues using structural foetal MRI.

MRI has the advantage over US in that sophisticated sequence design allows imaging beyond macromorphology. Most of these techniques have only found utility postnatally; however, scanner hardware advancements and postprocessing techniques covered in our review would facilitate the translation of these findings to foetal MRI. The current evidence from the postnatal literature appears to support the hypothesis that the abnormalities seen on dMRI are the consequence of the underlying hydrocephalus and not of foetal surgery. The use of dMRI would be justified for the characterisation of two distinct phenomena. First, supratentorial microstructural changes visible on dMRI can be related to hydrodynamic changes secondary to CSF flow obstruction at the level of the foramen. Second, the persisting hydrodynamic changes and the pathological stimuli to the ascending fibre pathways may alter the development of neuronal connectivity, which remains yet to be demonstrated in humans. There is emerging evidence that at least a part of the white matter damage in spina bifida would provide microstructural basis for neuropsychological abnormalities. Based on dMRI, the reconstruction of major cerebral and peripheric neuronal pathways are possible by tractography. Further methodological research may be necessary in order to improve the spatial resolution without further increasing the acquisition time and to match the tractography findings in spina bifida patients with their functional outcome.

MRS may detect metabolite concentrations that arise from leakage of cerebrospinal fluid through the skin defect present in spina bifida into the amniotic fluid. While MRS can be performed prenatally, these findings remain to be confirmed by MRS studies specifically focusing on spina bifida, and further research is needed to find out whether spina bifida is associated with different metabolite concentrations in the brain parenchyma.

Due to the expected benefits of early diagnostics and therapy in this highly sensitive patient population, spina bifida research has historically been on the forefront of advancing technology. The emerging MRI methods introduced in our review herald the beginning of an important endeavour of automated evaluation of foetal morphology and physiology free from observer bias. Ultimately, a priority would be to use MRI techniques that will increase the anatomical resolution and contrast without significantly prolonged imaging time and incorporate diagnostic information beyond morphology, which holds the key to develop novel imaging biomarkers in spina bifida.

## Data Availability

Imaging data that was used is available on request in a fully anonymised form.

## Abbreviations

ADC: Apparent diffusion coefficient
CSF: Cerebro-spinal fluid
dMRI: Diffusion magnetic resonance imaging
DTI: Diffusion tensor imaging
FA: Fractional anisotropy
HR: High resolution
MOMS: Management of myelomeningocele study
MRI: Magnetic resonance imaging
MRS: Magnetic resonance spectroscopy
SR: Super-resolution
SVR: Slice-to-volume reconstruction
VP: Ventriculoperitoneal shunting
